# Evaluation of glymphatic system activity using diffusion tensor image analysis along the perivascular space and amyloid PET in older adults with objectively normal cognition: a preliminary study

**DOI:** 10.3389/fnagi.2023.1221667

**Published:** 2023-07-27

**Authors:** Chae Jung Park, Sang-Young Kim, Jun Hwee Kim, Nak-Hoon Son, Jin Young Park, Yong Hyu Jeong, Hyun Jeong Kim, Jaesub Park, Woo Jung Kim

**Affiliations:** ^1^Department of Radiology, Yongin Severance Hospital, Yonsei University College of Medicine, Yongin-si, Gyeonggi-do, Republic of Korea; ^2^MR Clinical Science, Philips Healthcare Korea, Seoul, Republic of Korea; ^3^Department of Statistics, Keimyung University, Daegu, Republic of Korea; ^4^Institute of Behavioral Science in Medicine, Yonsei University College of Medicine, Seoul, Republic of Korea; ^5^Department of Psychiatry, Yongin Severance Hospital, Yonsei University College of Medicine, Yongin-si, Gyeonggi-do, Republic of Korea; ^6^Center for Digital Health, Yongin Severance Hospital, Yonsei University Health System, Yongin-si, Gyeonggi-do, Republic of Korea; ^7^Department of Nuclear Medicine, Yongin Severance Hospital, Yonsei University College of Medicine, Yongin-si, Gyeonggi-do, Republic of Korea; ^8^Department of Psychiatry, National Health Insurance Service Ilsan Hospital, Goyang-si, Gyeonggi-do, Republic of Korea

**Keywords:** subjective cognitive decline, glymphatic system, diffusion tensor imaging, magnetic resonance imaging, amyloid PET

## Abstract

**Objectives:**

Diffusion tensor image analysis along the perivascular space (DTI-ALPS) is a recently introduced method for the assessment of the glymphatic system without the need for contrast injection. The purpose of our study was to assess the glymphatic system in cognitively normal older adults with or without subjective cognitive decline (SCD) using DTI-ALPS, and correlating with amyloid PET.

**Design and participants:**

To evaluate the glymphatic system in cognitively normal older adults using DTI-ALPS, we built a prospective cohort including a total of 123 objectively cognitively normal older adults with or without SCD. The ALPS index was calculated from DTI MRI and was assessed by correlating it with standardized uptake value ratios (SUVRs) from amyloid PET and clinically relevant variables. The study subjects were also divided into amyloid “positive” and “negative” groups based on the result of amyloid PET, and the ALPS indices between those two groups were compared.

**Results:**

The ALPS index was not significantly different between the normal and SCD groups (*P* = 0.897). The mean ALPS index from the amyloid positive and amyloid negative group was 1.31 and 1.35, respectively, which showed no significant difference (*P* = 0.308). Among the SUVRs from variable cortices, that of the paracentral cortex was negatively correlated with the ALPS index (*r* = −0.218, *P* = 0.016). Multivariate linear regression revealed that older age (coefficient, −0.007) and higher SUVR from the paracentral cortex (coefficient, −0.101) were two independent variables with a significant association with a lower ALPS index (*P* = 0.015 and 0.045, respectively).

**Conclusion:**

DTI-ALPS may not be useful for evaluation of the glymphatic system in subjects with SCD. Older age was significantly associated with lower ALPS index. Greater amyloid deposition in the paracentral cortex was significantly associated with lower glymphatic activity in cognitively normal older adults. These results should be validated in future studies on the relationships between ALPS index and other fundamental compartments in glymphatic system, such as perivenous space and the meningeal lymphatic vessels.

## Introduction

The glymphatic system is a recently discovered waste drainage system in the brain that involves movement of cerebrospinal fluid (CSF) through the perivascular and interstitial spaces (Taoka and Naganawa, 2020). The perivascular space around the arteries allows CSF to enter the interstitial spaces through water channels controlled by aquaporin 4. CSF entering the interstitial space washes away waste proteins within the tissues, such as amyloid-β (Aβ) and metabolites, and facilitates the distribution of glucose, lipids, amino acids, and neuromodulators (Iliff et al., 2012, 2013; Jessen et al., 2015). Therefore, the glymphatic system is considered to play an important role in many neurological diseases including Alzheimer’s disease (AD), normal pressure hydrocephalus, sleep disorder, and vascular disease (Taoka et al., 2017; Jiang, 2019).

Evaluation of the glymphatic system has been performed with fluorescent tracers in animal experiments (Iliff et al., 2012, 2013; Jessen et al., 2015). As radiotracer studies are limited in the human population, many previous studies utilized magnetic resonance imaging (MRI) techniques to measure and visualize the CNS fluid flow compatible with the glymphatic system, primarily with gadolinium-based contrast agents (Lee M. et al., 2022). However, complex MRI acquisition including invasive intrathecal or intravenous contrast agent injection still limits widespread clinical application. Recently, a non-invasive method called “Diffusion Tensor Image-Analysis aLong the Perivascular Space” (DTI-ALPS) has been introduced for glymphatic assessment to overcome the drawbacks of contrast enhanced MRI (Taoka et al., 2017). With this method, the motion of water molecules in the direction of perivascular spaces can be evaluated by measuring diffusivity using the diffusion tensor method (Taoka et al., 2017). This DTI-based method has effectively shown altered glymphatic function in many neurological diseases (Bae et al., 2021; Chen et al., 2021; Steward et al., 2021; Kamagata et al., 2022; Lee D. et al., 2022; Ruan et al., 2022). Many previous studies have assessed, measured, and visualized normal CNS fluid flow compatible with the glymphatic hypothesis in human participants (Lee M. et al., 2022). However, few studies have applied DTI-ALPS for evaluation of glymphatic activity in non-diseased population.

Subjective cognitive decline (SCD) is the self-reported experience of impaired memory but with normal cognitive performance on an objective neuropsychological test (Jessen et al., 2014). Although the results of some studies are inconsistent, biomarker studies of SCD including neuroimaging generally suggest SCD as an early prodromal stage of AD that precedes mild cognitive impairment (MCI). This is based on the similar underlying pathological changes in SCD to those in MCI and dementia (Jessen et al., 2014; Parker et al., 2022). Based on previous studies that revealed an impaired glymphatic system in MCI and AD, we hypothesized that the glymphatic system would be decreased in subjects with SCD with normal cognition, and that DTI-ALPS may capture this impairment.

The purpose of the present study was to assess the glymphatic system in cognitively normal older adults with or without SCD using DTI-ALPS, which is an indirect measurement of the glymphatic system. For further understanding of the glymphatic system, we correlated DTI-ALPS results with amyloid PET and aimed to identify relevant clinical and imaging features showing significant associations with DTI-ALPS.

## Materials and methods

### Participants

This is a prospective cohort study of older adults without dementia recruited through advertisement in the community from September 2020 to April 2021. The inclusion criteria were as follows: (1) age 65–79 years; (2) normal cognitive profiles on a comprehensive neuropsychological battery; and (3) normal range of activities of daily living (ADL). Exclusion criteria were as follows: (1) illiteracy; (2) severe impairment in auditory function or vision causing difficulty in communication; (3) previous history of major neurological (Parkinsonism, epilepsy, stroke, and head trauma) or psychiatric illness (schizophrenia, bipolar disorder, and major depression); (4) abnormal brain MRI findings including hemorrhage, infarctions, and other space-occupying lesions; and (5) difficulties in MRI acquisition (claustrophobia or non-removable ferromagnetic implants). The study also excluded subjects considered to have dementia or MCI at the time of screening according to the National Institute on Aging Alzheimer’s Association criteria (Albert et al., 2011). Finally, a total of 123 older adults comprised the prospective cohort of study subjects. Study subjects with objectively normal cognition were allocated to either older adults with or without SCD based on the results of a self-reported questionnaire to measure self-perceived cognitive decline. Institutional Review Board of Yongin Severance Hospital approved this study (9-2020-0080).

### Assessment of cognitive function and SCD

Objective cognitive function was measured through the Seoul Neuropsychological Screening Battery-Core (SNSB-C) (Jahng et al., 2015). The SNSB-C consists of 14 tasks evaluating five cognitive domains: attention, language, visuospatial function, memory, and fronto-executive function, including the Mini-Mental State Examination (MMSE). The results of SNSB-C were used only to determine whether cognitive function was normal, although MMSE scores were used for regression analysis in this study.

Among 123 subjects with objectively normal cognition, the following two scales were used to determine whether SCD was present. The Subjective Cognitive Decline Questionnaire (SCD-Q) (Rami et al., 2014) consists of 24 yes or no questions assessing the difficulty of activities requiring cognitive function in the recent 2 years, with a score ranging from 0 to 24. The Memory Age-associated Complaint Questionnaire (MAC-Q) (Crook et al., 1992) is comprised of a six-item scale evaluating subjective age-related memory decline compared with memory at younger ages, and ranges from 0 to 35, with a higher score indicating more subjective memory decline. On both scales, a higher score means higher subjective memory complaints. In this study, subjects with an SCD-Q score of seven or higher and an MAC-Q score of 25 or higher were classified as the SCD group, otherwise, they were classified as the normal group.

### MRI acquisition and preprocessing

Magnetic resonance imaging data were acquired using a 32-channel 3T MRI scanner (Ingenia Elition X or Ingenia CX, Philips Healthcare, Best, Netherlands). The 3D T1-weighted images and DTI data were preprocessed for robust estimations of glymphatic activity. Details regarding MRI acquisitions and preprocessing methods can be found in the Supplementary material 1 and Supplementary Figure 1.

### Calculation of the ALPS index

**FIGURE 1:**
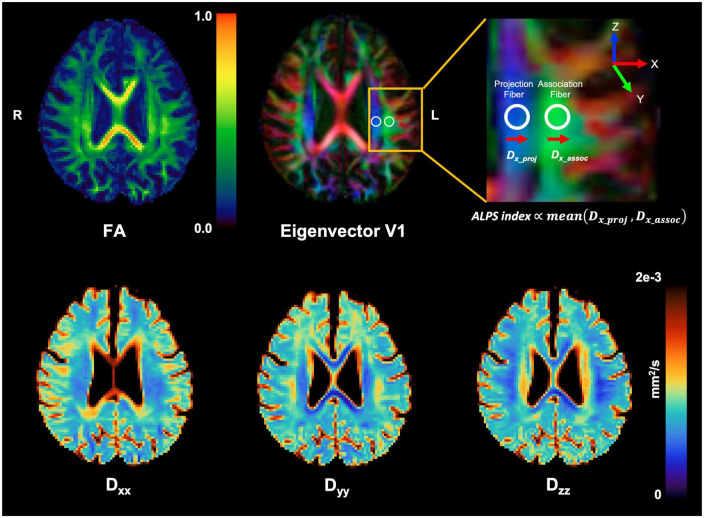
Schematic drawing of diffusivity measurement using the DTI-ALPS methods. Reconstructed diagonal element maps (Dxx, Dyy, and Dzz) of the diffusion tensor and its derived FA and first eigenvector (V1) maps. Regions of interest (ROIs) for calculation of ALPS index were manually placed at the label of the lateral ventricle on a color-coded FA map **(top middle)**. For better visualization for ROI placement, the target brain region (yellow box) was magnified **(top right)**. Note that the ALPS index is proportional to the amount of diffusivity perpendicular to projection and association fibers.

The method for DTI-ALPS processing and measurement was adopted from a previous publication (Taoka et al., 2017). The details are explained in Figure 1.

Briefly, on the diffusivity maps, one neuroradiologist (CP with 5 years’ experience) blinded to the clinical findings measured (1) the diffusivities along the *x*-axis (Dx) in the projection (Dxxproj), the association (Dxxassoc), and the subcortical (Dxxsubc) neural fiber areas, (2) the diffusivities along the *y*-axis (Dy) in three neural fiber areas (Dyyproj, Dyyassoc, and Dyysubc), and (3) the diffusivities along the *z*-axis (Dz) in three neural fiber areas (Dzzproj, Dzzassoc, and Dzzsubc). According to a previous study (Taoka et al., 2017), Dxxproj and/or Dxxassoc express water diffusion along the perivascular space without interference from neural fibers, reflecting glymphatic activity. In contrast, Dxxsubc would not reflect pure perivascular water diffusion since the subcortical neural fibers pass parallel to the perivascular direction obscuring glymphatic diffusion. In addition, Dy and Dz lay perpendicular to the perivascular direction and would also not reflect glymphatic diffusion.

The ALPS index was calculated for individual subjects to evaluate the activity of the glymphatic system. This index is provided as the ratio of two sets of diffusivity values that are perpendicular to dominant fibers in the tissue, that is, the ratio of mean of Dxxproj and Dxxassoc to the mean of Dyyproj and Dzzassoc is as follows.


A⁢L⁢P⁢S⁢i⁢n⁢d⁢e⁢x=m⁢e⁢a⁢n⁢(D⁢x⁢x⁢p⁢r⁢o⁢j,D⁢x⁢x⁢a⁢s⁢s⁢o⁢c)/m⁢e⁢a⁢n⁢(D⁢y⁢y⁢p⁢r⁢o⁢j,D⁢z⁢z⁢a⁢s⁢s⁢o⁢c).

The ALPS index is close to one when the perivascular water diffusion is minimal but gets larger with increased perivascular diffusivity. The ALPS-indices were obtained from both cerebral hemispheres, and those two ALPS indices were averaged and used for further analysis.

### The ε4 allele of the apolipoprotein E genotyping

The apolipoprotein E (ApoE4) is located on chromosome 19 and is associated with the production of amyloid, and has been consistently shown to be overrepresented in patients with AD in numerous population-based genetic studies. In order to discriminate the genetic risk of incident AD (Müller-Gerards et al., 2019), all subjects were categorized as ApoE4 positive if they had at least one copy of the ApoE4 and as ApoE4 negative otherwise. The ApoE genotype was confirmed using the polymerase chain reaction.

### Amyloid PET acquisition, preprocessing, and image analysis

Brain Aβ deposition was visualized by amyloid PET using tracer [18F] flutemetamol and quantified as the standardized uptake value ratio (SUVR) of each cortical region (Barthel et al., 2011; Thal et al., 2015). Processing was performed using the SPM12^1^ with PETPVE12 toolbox^2^ derived from a large-scale amyloid staging study using the partial volume effect (PVE) correction method. PET scans were rigidly co-registered to the corresponding structural MRI scans. The inverse warping parameters identified through spatial normalization of the subjects’ MRI scan to an MNI-152 T1-weighted template provided by the PETPVE toolbox (Gonzalez-Escamilla et al., 2017). Then, for subsequent analyses using relatively small regions of interest, corrections for PVEs were performed using the Muller-Gartner method (Müller-Gärtner et al., 1992). Regional PET uptake values were sampled from 82 brain regions defined in the Desikan-Killany atlas (Desikan et al., 2006) and converted to MNI space. The atlas labels were multiplied by a binary gray matter mask of the reference template threshold at 50% gray matter probability and were propagated to the subjects’ native space using each subject’s inverse deformation field. Means of regional PET uptake were converted to SUVR by scaling to the mean uptake of the whole cerebellum.

Amyloid PET positivity was classified using the Centiloid standard pipeline. Briefly, the Centiloid standard SUVR of the standard global cortical target volume of interest (VOI) was obtained using the whole cerebellum VOI as the reference region, and the Centiloid unit (CL) was calculated. If this value was greater than the threshold value, the subject was classified as amyloid PET positive. Detailed methods and validation are described in Supplementary material 2.

### Statistical analysis

Statistical analysis was performed using R software (version 3.5.1). *P* < 0.05 was considered to indicate a statistically significant difference; *P*-values were two-sided. As the ALPS index was obtained from both the right and left sides in each participant, the average value of those two indices was used for statistical analysis.

The demographic findings, diffusivities, ALPS index, and SUVRs from amyloid PET in asymptomatic adults and adults with SCD were compared using the Student’s *t*-test, Mann–Whitney test, and Chi-square test. The study subjects were divided into “amyloid positive” and “amyloid negative” groups based on a CL score of 26, and the ALPS indices between those two groups were compared using two sample *t*-test. The correlation between the ALPS index and SUVRs from variable cortical regions acquired from PET were evaluated using Spearman’s correlation coefficient. A simple linear regression was used to examine the association of ALPS index with other clinically relevant variables (age, sex, duration of education, MMSE scores, ApoE4, and presence of SCD). ApoE4 was dichotomized as one (for ApoE4 positive) or zero (for ApoE4 negative). Then, the variables that were proven to have significant association with the ALPS index were integrated into a multivariate linear regression model.

## Results

### Study participants

The baseline clinical characteristics of the 123 participants are summarized in Table 1. Among the total 123 subjects with objectively normal cognition, 63 adults were asymptomatic, and 60 adults were categorized as SCD. Years of education and cognitive performance were not significantly different between adults with and without SCD. The scores of SCD-Q and MAC-Q were significantly higher in subjects with SCD compared to asymptomatic adults.

**TABLE 1:** Baseline clinical characteristics of study participants.

Clinical variables	Without SCD (*n* = 63)	With SCD (*n* = 60)	*P*-value*
Age (years)	73.0 ± 4.2	73.7 ± 3.5	0.316
Female, no. (%)	43 (68.3%)	41 (68.3%)	>0.999
Education (years)	10.6 ± 4.8	9.5 ± 4.3	0.181
**Cognitive performance**			
K-MMSE (/30)	26.7 ± 2.3	27.1 ± 1.9	0.300
SNSB-C total (*Z* score)	−0.05 ± 1.08	0.07 ± 0.91	0.518
SCD-Q	4.7 ± 3.7	11.7 ± 3.5	<0.001
MAC-Q	24.6 ± 2.7	28.5 ± 2.6	<0.001
ApoE4			0.352
No	53	50	
Yes	10	13	

Values are expressed as mean ± standard deviation or number (percentage).

*Calculated from either Student’s t-test or Mann–Whitney test for continuous variables and from Chi-square test for categorical variables.

SCD, subjective cognitive decline; K-MMSE, the Korean version of the Mini-Mental State Examination; SCD-Q, Subjective Cognitive Decline Questionnaire; MAC-Q, Memory Age-associated Complaint Questionnaire; ApoE4, ε4 allele of the apolipoprotein E.

### Comparison of the DTI-ALPS and amyloid PET findings between adults with and without SCD

The diffusivities and ALPS index were compared between cognitively normal adults with and without SCD (Table 2). There were no significant differences regarding the diffusivities and ALPS index between the two groups. Regarding amyloid PET findings, the adults with SCD showed a tendency to present higher SUVR values compared to those from asymptomatic adults in all regions, however, there were no significant differences (Supplementary Table 1).

**TABLE 2:** Comparison of diffusivity and ALPS index between cognitively normal older adults with and without subjective cognitive decline (SCD).

Diffusivity	Without SCD	With SCD	*P*-value*
Dxxproj	0.577 (0.469–0.781)	0.577 (0.431–0.744)	0.675
Dxxassoc	0.664 (0.536–0.842)	0.677 (0.500–0.868)	0.968
Dyyproj	0.441 (0.323–0.640)	0.454 (0.323–0.593)	0.383
Dyyassoc	0.923 (0.710–1.183)	0.946 (0.659–1.162)	0.658
Dzzproj	1.149 (0.888–1.488)	1.112 (0.876–1.418)	0.401
Dzzassoc	0.465 (0.318–0.744)	0.462 (0.333–0.811)	0.761
ALPS index	1.328 (1.040–1.161)	1.336 (0.943–1.643)	0.897

Values are shown as median (range).

*P-values were calculated from the Student’s *t*-tests.

Diffusivity was measured with apparent diffusion coefficient values (×10^–3^ mm^2^/s).

### Comparison of ALPS indices between amyloid positive/negative groups

Study subjects were divided into 11 amyloid positive and 112 amyloid negative based on a CL score of 26. The mean ALPS index from the amyloid positive and amyloid negative group was 1.31 and 1.35, respectively, and there was no statistically significant different between those two values (*P* = 0.308).

### Correlation between the ALPS index and SUVR from PET

**FIGURE 2:**
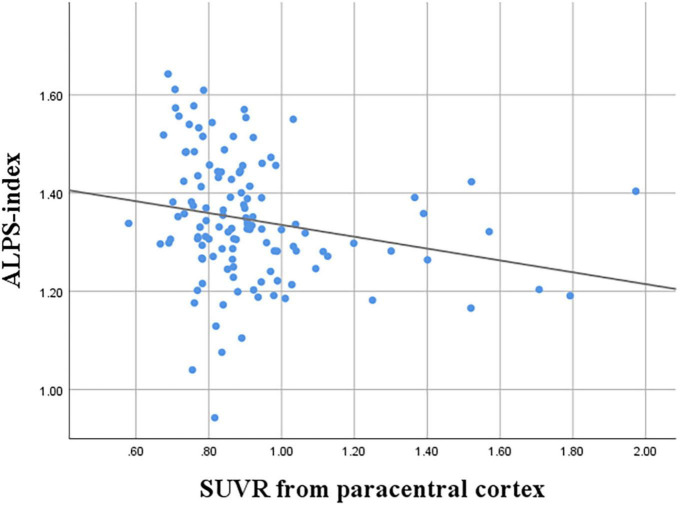
Correlation between ALPS index and SUVR of the paracentral cortex (*r* = −0.218, *P* = 0.016).

The correlation between the ALPS index and SUVRs from variable cortical regions were evaluated (Table 3). Among the SUVRs from variable cortices, the SUVR from the paracentral cortex was the only variable that was negatively correlated with the ALPS index (*r* = −0.218, *P* = 0.016) (Figure 2).

**TABLE 3:** Correlation analysis between the ALPS index and SUVR from amyloid PET.

Cortical regions	SUVR	Correlation coefficient	*P*-value*
Caudal anterior cingulate	0.952 ± 0.244	−0.156	0.084
Caudal middle frontal	0.990 ± 0.256	−0.148	0.103
Frontal pole	0.922 ± 0.402	−0.108	0.236
Inferior parietal	1.102 ± 0.277	−0.098	0.283
Inferior temporal	1.091 ± 0.202	−0.111	0.222
Lateral orbito-frontal	1.017 ± 0.237	−0.072	0.430
Middle temporal	1.064 ± 0.231	−0.092	0.310
Paracentral	0.918 ± 0.227	−0.218	0.016*
Pars opercularis	0.987 ± 0.254	−0.157	0.083
Pars orbitalis	1.100 ± 0.299	−0.131	0.148
Pars triangularis	1.064 ± 0.311	−0.165	0.069
Postcentral	0.978 ± 0.160	−0.025	0.780
Precentral	0.894 ± 0.151	−0.030	0.740
Precuneus	0.992 ± 0.296	−0.124	0.170
Rostral anterior cingulate	0.956 ± 0.239	−0.038	0.677
Rostral middle frontal	0.987 ± 0.338	−0.139	0.125
Superior parietal	1.039 ± 0.242	−0.159	0.078
Superior temporal	1.023 ± 0.167	−0.132	0.145
Supra-marginal	1.073 ± 0.256	−0.132	0.144

Values are shown as mean ± standard deviation.

*P-values were calculated from the Spearman’s correlation analysis.

### Multivariate linear regression analysis of ALPS index

A simple linear regression analysis was performed for each clinical variable – age, sex, duration of education, MMSE scores, ApoE4 status, and the presence of SCD (either asymptomatic or diagnosed as SCD) – to assess whether it had significant association with the ALPS index. Age, duration of education, MMSE scores, and ApoE4 status were significantly associated with the ALPS index. The presence of SCD was not significantly related to the ALPS index. Those four clinically relevant variables were then entered into the multivariate regression analysis together with the SUVR from the paracentral cortex, which was the only variable that had significant association with DTI-ALPS among SUVRs from the cortical regions. As a result, age (coefficient, −0.007, *P* = 0.015) and SUVR from the paracentral cortex (coefficient, −0.101, *P* = 0.045) were the two variables that revealed a significant negative correlation with the ALPS index (Table 4).

**TABLE 4:** Multiple linear regression analysis of ALPS index.

Variable	Coefficients	*P*-value*
Age	−0.007 (−0.013 to −0.002)	0.015
Duration of education	−0.002 (−0.007 to 0.004)	0.558
MMSE scores	0.004 (−0.008 to 0.016)	0.482
ApoE4	0.035 (−0.024 to 0.094)	0.238
SUVR from paracentral cortex	−0.101 (−0.202 to −0.002)	0.045

Values in parentheses are 95% confidence intervals.

*P-values were calculated from a multivariate regression analysis.

## Discussion

This study aimed to evaluate the glymphatic system in cognitively normal older adults using non-invasive DTI by correlating it with clinical variables and amyloid PET. There were no significant differences in DTI-ALPS indices between adults with or without SCD. The greater amyloid burden in the paracentral cortex and older age were significantly associated with a lower ALPS index, indicating impaired glymphatic clearance in cognitively normal older adults.

Intrathecal or intravenous contrast injection for evaluation of the glymphatic system is invasive and poses risks for gadolinium deposition in the brain (Öner et al., 2017). Therefore, the DTI-ALPS method was recently developed utilizing non-invasive DTI without need for a contrast agent for assessment of the glymphatic system (Taoka et al., 2017). As DTI can be rapidly obtained while allowing multiple image acquisitions in a single subject, it enables monitoring of the status of the glymphatic activity over time. DTI-ALPS has been applied in neurodegenerative diseases including AD (Steward et al., 2021) and Parkinson’s disease (Chen et al., 2021), and the greater extent of impaired glymphatic clearance was noted in patients with more severe degrees of cognitive decline. Glymphatic function assessed with DTI-ALPS was also impaired in patients with normal pressure hydrocephalus (Bae et al., 2021), Parkinson’s disease (Ruan et al., 2022), and isolated REM sleep behavior disorder (Lee D. et al., 2022) compared to normal controls. Therefore, DTI-ALPS may be a feasible, non-invasive tool for the evaluation of the glymphatic system. In this study, we evaluated whether cognitively normal older adults who complained of SCD showed a significantly impaired glymphatic system compared to asymptomatic adults. However, there were no significant differences between diffusivities or ALPS index between adults with and without SCD. As SCD is self-experienced decline in cognitive function without evidence of objective cognitive impairment, the degree of impairment in glymphatic clearances may be too subtle to be captured in the ALPS index. Therefore, DTI-ALPS may not be useful as an indirect measurement of glymphatic activity in cognitively unimpaired adults with SCD. Future studies with larger number of adults with SCD may be needed for evaluation of impairment in glymphatic clearances.

This study investigated the correlation between amyloid deposition and the ALPS index, and found that the amyloid deposition, particularly in the paracentral cortex, was significantly associated with a lower ALPS index. ALPS index, which is the measurement of diffusivity along the direction of perivascular spaces, has been regarded as an indirect measurement of glymphatic activity as the main stream of glymphatic system involves movement of CSF along the perivascular spaces. Meanwhile, the key concept of glymphatic system is that after subarachnoid CSF enters the brain through periarterial spaces and mixes with interstitial fluid, it drains through perivenous spaces, meningeal lymphatic vessels (MLVs), or perineural pathways and finally reach at the deep cervical lymph nodes (Zhang et al., 1990; Iliff et al., 2013; Jessen et al., 2015; Taoka et al., 2017; Naganawa et al., 2020). As all these key elements in the glymphatic system correlate and regulate each other significantly, alteration in one component either due to aging or neurodegenerative diseases can affect the remainder profoundly. MLVs are one of the main routes for CSF efflux in the glymphatic system (Louveau et al., 2017; Jiang et al., 2022), and is well-known that these vessels experience functional decline with aging (Da Mesquita et al., 2021). Several previous studies with MRI reported their dorsal distribution along the venous sinuses that they are around almost all dural venous-parasagittal structures (Kuo et al., 2018; Albayram et al., 2022). Several recent studies with mice have suggested that either deterioration or ablation of MLVs results in increased amyloid deposition, specifically along the dura adjacent to the superior sagittal sinus (Da Mesquita et al., 2018; Wang et al., 2019). One study also reported significant amyloid deposition in the dura mater adjacent to the superior sagittal sinus in AD patients compared to normal controls (Da Mesquita et al., 2018). The authors concluded that prominent meningeal amyloid deposition occurs in AD patients and in mouse models of AD after MLV ablation. Even though our study subjects did not include patients with dementia, it can be inferred from previous studies that damage of function in MLVs, either due to aging or neurodegenerative diseases, can result in subsequent amyloid deposition in the adjacent structures along the superior sagittal sinus. As each compartment of glymphatic system is closely correlated with each other (Da Mesquita et al., 2018), impaired drainage through MLVs may affect overall glymphatic activity at the level of perivascular spaces, which can be captured by decreased ALPS index. Or, conversely, impaired glymphatic activity at the level of perivascular spaces may in turn affects the function of MLVs, which may attribute to the greater amyloid burden in the parasagittal regions including paracentral cortex. The reason why the specific paracentral cortex, rather than other regions along the superior sagittal sinus, showed significant association with ALPS index is yet elucidated. The reason for the lack of association of amyloid deposition in the posterior cingulate cortex or medial orbitofrontal cortex, where frequent amyloid deposits are encountered in AD, with DTI-ALPS is also not clear. Future studies with larger subjects including patients with objective cognitive decline may give insight regarding the association between the indirect measurement of glymphatic activity, i.e., ALPS index and amyloid deposition in particular brain regions. We believe that our study has its novelty as this is the first study that correlated the ALPS with amyloid PET findings in cognitively normal older adults.

This study found that age was the only clinical variable that was negatively correlated with the ALPS index. Similar results were observed in several previous studies that evaluated the impact of clinical demographics and vascular risk factors on the ALPS index in healthy subjects (Zhou et al., 2020; Zhang et al., 2021). During aging, the number of polarized aquaporin-4 channels on the end feet of astrocyte cells and CSF production decrease along with arterial pulsatility, attributed to the drop of glymphatic function (Jessen et al., 2015). In one recent publication, the ALPS index was also lower in males and patients with hypertension (Zhang et al., 2021). However, the ALPS index was not affected by these variables in the present study. These inconsistent findings may be attributed to different demographic characteristics, as the study subjects were older than those of the previous study (Zhang et al., 2021) (mean age 73.4 vs. 60.8 years) and had a higher proportion of adults with hypertension (45.6 vs. 39.4%). A larger number of normal older adults recruited from heterogeneous backgrounds is needed to evaluate the impact of clinical variables on the ALPS index.

A previous study, which first introduced the DTI-ALPS method for the evaluation of glymphatic activity, reported a significant positive correlation between the ALPS index and MMSE scores, indicating lower water diffusivity along the perivascular space in relation to AD severity (Taoka et al., 2017). Another study also reported significant correlations between DTI-ALPS and MMSE after adjustment of clinically relevant variables (Steward et al., 2021). However, the current study did not identify any significant correlation between MMSE scores and the ALPS index. As it included only adults with objectively normal cognition, excluding patients with documented cognitive decline, the differences in MMSE scores among subjects might be subtle. Most enrolled subjects from previous studies were patients with variable degrees of cognitive decline, demonstrating significantly lower MMSE scores compared to those in the present study.

According to the specific cut-off of SUVR from amyloid PET, 112 and 11 study subjects were classified into amyloid negative and positive group, respectively. As we have only enrolled elderly adults without objective cognitive decline, the number of subjects who had amyloid positivity was inevitably small. We observed that there was no statistically significant difference in ALPS index between the two groups, however, future studies with larger number of study subjects should validate our study results. In addition, further studies including patients with documented cognitive decline may give insight with regard to association between ALPS index and amyloid positivity.

Our study had several limitations. First, we only enrolled cognitively normal older adults with and without SCD and excluded patients with objective cognitive decline. The relationship between cognitive scores and the ALPS index may be reliably assessed in future studies including larger numbers of both cognitively normal and cognitively impaired adults. Second, as we performed cross-sectional analysis, it was not possible to examine the clinical significance of amyloid accumulation in cognitively normal individuals. In fact, there have been inconsistencies regarding the significance of amyloid deposition on future cognitive decline in the cognitively normal populations (Chételat et al., 2013; Guo et al., 2020). In addition, there is no established cutoff for SUVR from each cortical region that determines the significance of amyloid deposition. The SUVRs were correlated with the ALPS index from all cortical regions in a form of continuous variables. In this way, a specific cortical region was identified that may have an impact on the glymphatic system. Third, the DTI-ALPS was only used for assessment of glymphatic activity, and contrast-enhanced imaging methods were not performed in this study. Contrast-enhanced MRI may be superior to non-contrast MRI for glymphatic imaging, as it provides objective measurement of the degree of glymphatic flow changes (Lee M. et al., 2022). However, acquisition of contrast-enhanced sequences in normal individuals was not ethical. Despite these limitations, the researchers believe that DTI-ALPS is a feasible method for *in vivo* assessment of the glymphatic system, as demonstrated in many previous studies. Fourth, we were not able to include other behavioral risk factors for dementia (e.g., exercise, sleep disturbances, or social activity) in our study. The associations between ALPS index and other dementia risk factors should be studied for evaluation of clinical relevance of DTI-ALPS.

## Conclusion

In conclusion, DTI-ALPS may not be useful for the evaluation of the glymphatic system in subjects with SCD. Older age was significantly associated with lower ALPS index. Greater amyloid deposition in the paracentral cortex was significantly associated with lower glymphatic activity in cognitively normal older adults. These results should be validated in future studies on the relationships between ALPS index, perivenous space, and the meningeal lymphatic drainage system.

## Data availability statement

The datasets presented in this article are not readily available because unable to seek consent from research subjects for external disclosure of original data. Requests to access the datasets should be directed to WJK, woojungkim@yuhs.ac.

## Ethics statement

The Institutional Review Board of Yongin Severance Hospital approved this study (9-2020-0080). The patients/participants provided their written informed consent to participate in this study.

## Author contributions

CJP performed the conceptualization, formal analysis, and visualization of data and wrote and revised the manuscript. S-YK performed the preprocessing for DTI-ALPS analysis and calculated the ALPS index. JHK supported the formal analysis and visualization of data. N-HS contributed to the process of statistical complementation in the major revision process. JP contributed to the grouping of subjects and fully analyzed the PET imaging data and major revision. JYP supported the research progress through additional funding and supervised the whole project. YHJ and HJK contributed to the acquisition of PET imaging data. WJK performed the major roles in conceptualization, supervision, writing the manuscript, data curation, funding acquisition, and project administration. All authors had approved the submitted version of the manuscript.

## Footnotes

1
http://www.fil.ion.ucl.ac.uk/spm

2
https://github.com/GGonEsc/petpve12
