# Supplementary material for: Evaluation of glymphatic system activity using diffusion tensor image analysis along the perivascular space and amyloid PET in older adults with objectively normal cognition: a preliminary study
Source: Front Aging Neurosci. 2023 Jul 27;15:1221667. doi: 10.3389/fnagi.2023.1221667 (PMC10413261; doi:10.3389/fnagi.2023.1221667)
Supplement: Supplementary file 1 [file Presentation_1.pdf]

Supplementary material 1.

MRI acquisition

MRI data were acquired using a 3T MRI scanner (Ingenia Elition X or Ingenia CX, Philips Healthcare, Best, Netherlands) with a 32-channel head coil. Using single-shot echo-planar imaging, diffusion tensor imaging (DTI) was performed in the axial plane using b-values of 0 and 1,000 s/mm^2 with 32 directions of diffusion gradients. Other imaging parameters for DTI were as follows: repetition time (TR) = 9900 msec; echo time (TE) = 77 msec; slice thickness = 2 mm; flip angle = 90º, field-of-view (FOV) = 224×224 mm^2; acquisition matrix = 112×112 mm^2.

The routine protocols were as follows: sagittal three-dimensional (3D) T1-weighted imaging (T1WI) with axial and coronal reconstruction, sagittal 3D fluid-attenuated inversion recovery (FLAIR) imaging with axial reconstruction, and 3D axial T2-weighted imaging (T2WI) and susceptibility-weighted imaging (SWI). The imaging parameters were as follows: 1) 3D T1WI: TR = 4.5 msec; TE = 2.0 msec; slice thickness = 1.0 mm; flip angle = 8º; acquisition matrix = 240×240; FOV = 200×200 mm^2; 2) 3D FLAIR: TR = 4800 msec; TE = 297 msec; slice thickness = 1.0 mm; flip angle = 90º; FOV = 200×200 mm^2; 3) 3D T2WI: TR = 2500 msec; TE = 240 msec; slice thickness = 2 mm; flip angle = 90º; FOV = 250×250 mm^2; 4) SWI: multi-echo fast-field-echo sequence, TR = 51 msec; total 6 echoes; first TE = 0 msec; echo interval = 6.0 msec; slice thickness = 2 mm; flip angle = 20º; FOV = 230×230 mm^2.

MRI preprocessing

The preprocessing of anatomical 3D T1WI data started with bias field correction using ANTs N4 algorithm followed by registration-based brain extraction (1). Subsequently, T1WI was aligned to the MNI space template using rigid-body registration, which enabled

the image to be aligned at the same orientation as the template (i.e., AC-PC alignment) without changing the original brain size and shape. Then, the AC-PC-aligned T1WI was registered to MNI space with symmetric image normalization (SyN) for maximizing the cross-correlation within the space of diffeomorphic maps (2). The output transforms were combined with the one obtained from AC-PC alignment to produce the final registration warp field. Last, the final warp field was applied to bias field-corrected T1WI for registration.

The DTI data were preprocessed with a 3D non-local mean filter to remove the Rician noise. Next, the b=0 image was used for brain extraction to obtain a brain mask. A noise-removed DTI dataset was corrected for eddy-current-induced distortions and subject movements using the FSL eddy tool (https://fsl.fmrib.ox.ac.uk/fsl/fslwiki/eddy) (3). However, we were not able to correct susceptibility-induced geometric distortion as phase-encoding reversed DTI data were not available in routine clinical trials. Eddy-current-corrected DTI data were then registered to an AC-PC-aligned T1W image resampled to 1.5 mm isotropic resolution. Finally, 6 elements of a diffusion tensor matrix were obtained by fitting DTI data using a Python library for analysis of diffusion MRI (DIPY, https://dipy.org/) (4), and maps of fractional anisotropy (FA), mean diffusivity (MD), and axial and radial diffusivity (AD and RD) were generated from the eigenvalues of the tensor.

Supplementary Figure 1. Schematic presentation of the MRI preprocessing.

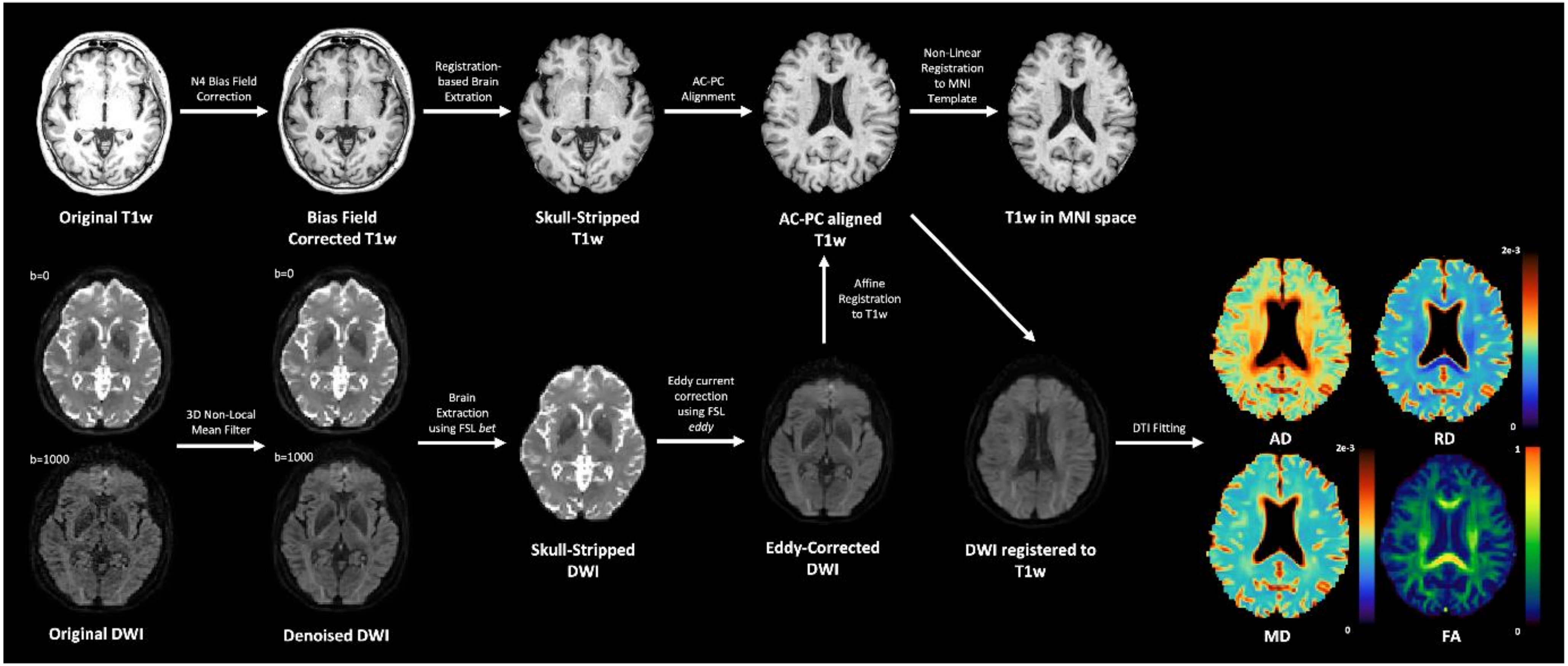

Supplementary material 2.

Detailed process of amyloid PET analysis using a partial volume effect correction (PVEc) pipeline

Imaging data were processed using statistical parametric mapping (SPM12, Wellcome Trust Center For Neuroimaging) and the PETPVE12 toolbox (https://github.com/GGonEsc/petpve12) implemented in Matlab R2021(MathWorks, Natick, MA).

MRI image processing

MRI scans were automatically segmented into gray matter (GM), white matter (WM), and cerebrospinal fluid (CSF) partitions using the segmentation function of PVEPET12 adopted from the VBM8 toolbox (http://dbm.neuro.uni-jena.de/vbm/). The partitions of each subject in native space were registered to an MNI-152 T1-weighted template provided by the PETPVE12 toolbox. In this process, two files containing individual reverse normalization parameters (deformity fields) were produced for imaging analysis.

PET image processing

Each subject's amyloid PET scans were co-registered to a bias-corrected image of the corresponding structured MRI scan using the PETPVE12 function, and visual inspection was performed. Correction for PVE followed the algorithm proposed by the Muller-Gartner method (PVEc-MG method) (5), which was implemented in the PETPVE12 toolbox. The PVEc-MG method is a three-compartment PVEc method that discriminates signals from brain GM, WM, and CSF and is one of the most widely used MRI-based methods for PET image analysis (6). In brief, this method assumes that the observed PET signal of a GM voxel is a spatially weighted average of the actual tracer uptake signal at the GM voxel and the

signal from surrounding WM and CSF. Spatial weights are determined by the point spread function (PSF) of the PET scanner. The proposed PVEc algorithm consists of correction for the spill-out effect of signal leakage from the GM to the surrounding tissue as well as the spill-in effect from the surrounding tissue to the GM compartment. Tracer activities in WM and CSF are assumed to be homogeneous in each compartment.

Extraction of regional SUVR

Regional amyloid PET uptake was sampled from 82 brain regions defined in the Desikan-Killiany atlas (7) (atlas included in the PETPVE12 toolbox, the original brain atlas was propagated to the MNI space). The Desikan-Killiany atlas covers the whole cerebral cortex and widely is used for amyloid PET studies including staging (8). The atlas labels were multiplied with the reference template's binary GM mask thresholded at 50% GM probability. The atlas in reference space was transformed into each subject's native space using inverse deformity fields. Mean uptake value of the whole cerebellum was extracted from the PVE-uncorrected PET image and used as a reference region, like in previous studies (9-12). The uptake value of voxels was converted to standard uptake value ratios by scaling to the mean uptake of the whole cerebellum in non-PVE-corrected data. The regional mean standardized uptake ratios of subjects were obtained using the function implemented in the PETPVE12 toolbox.

Standardization and validation of a local Centiloid standard pipeline

The standardization and validation of the PET imaging analysis methods are based on a dataset and standardized cortical and whole cerebellar volume of interest (VOI) templates, freely available on the Global Alzheimer Association Interactive Network website (GAAIN; http://www.gaain.org). This dataset was used for flutemetamol Centiloid scaling (13) and

consisted of a total of 74 subjects comprising 24 young controls, 20 AD patients, 20 psychotic MCI patients, and 10 elderly normal controls. All subjects underwent both Pittsburgh compound-B (PiB) and flutemetamol (Flute) scans.

Validation of the local Centiloid standard pipeline

We created the local standard Centiloid pipeline based on the details of a standard processing system provided by Klunket et al., (14) using SPM8 (Statistical Parametric Mapping, Version 8, Wellcome Trust Center for Neuroimaging, http://www.fil.ion.ucl.ac.uk) implemented in Matlab R2018 (MathWorks, Natick, MA). For processing, PET and T1 3D MRI images were normalized to MNI-152 space, and standard cortical and whole cerebellar VOI templates were applied. For validation, the SUVR values of local Centiloid standard pipeline were compared with the published SUVR values of standardization studies. As the Centiloid unit (CL) calculation formula, the previously published formula was used as follows:

$$CL = 93.72 \text{ x } SUVR_{PiB} - 94.56 \text{ (published)} \quad (1)$$

Differences between published SUVR values and local standard Centiloid SUVR values for each subject were all less than 5%. All values were correlated with each other: Local standard Centiloid $SUVR_{PiB} = 0.997 \text{ x } SUVR_{published\text{-}PiB} + 0.003$ ($R^2 = 1.000$): Local standard Centiloid $SUVR_{Flute} = 0.998 \text{ x } SUVR_{published\text{-}Flute} + 0.002$ ($R^2 = 0.999$) (Figure 1A, 1B). Using published equation (1), local CL was calculated and was correlated with Published CL: Local CL = 0.99 × Published CL – 0.17 ($R^2 = 1.000$) (Figure 1C). These results meet the acceptance criteria of the Centiloid standard (slope between 0.98 and 1.02, intercept between −2 and 2 CL, and R^2 >0.98).

Figure 1. Correlation between standard Centiloid SUVRs, CL, and published values.

Abbreviations: Flute, flutemetamol; PiB, Pittsburgh compound-B.

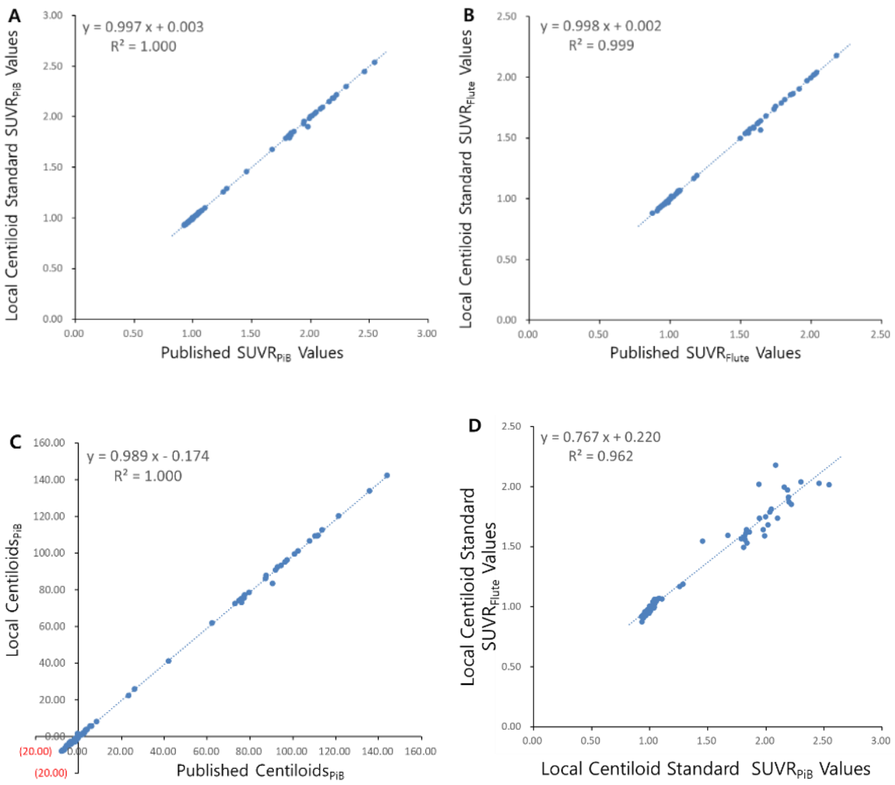

We obtained the correlation between local standard Centiloid $SUVR_{PiB}$ and local standard Centiloid $SUVR_{Flute}$, and the result ($y = 0.77x + 0.22$, R^2 of 0.96) was identical to the published data (Figure 1D). The following conversion equation was calculated.

$$CL = 121.42 \text{ x } SUVR_{Flute} - 121.16 \quad (2)$$

Supplementary Table 1. Comparison of SUVRs between older adults with and without SCD

SUVR	without SCD	with SCD	P value
Caudal anterior cingulate	0.92 ± 0.19	0.99 ± 0.29	0.088
Caudal middle frontal	0.95 ± 0.20	1.03 ± 0.30	0.080
Frontal pole	0.88 ± 0.34	0.97 ± 0.46	0.242
Inferior parietal	1.07 ± 0.23	1.14 ± 0.32	0.149
Lateral orbitofrontal	0.99 ± 0.19	1.04 ± 0.27	0.218
Middle temporal	1.04 ± 0.21	1.09 ± 0.25	0.288
Paracentral	0.90 ± 0.18	0.94 ± 0.27	0.348
Pars opercularis	0.95 ± 0.19	1.02 ± 0.30	0.139
Pars orbitalis	1.06 ± 0.25	1.14 ± 0.34	0.134
Pars triangularis	1.02 ± 0.24	1.11 ± 0.37	0.110
Postcentral	0.96 ± 0.13	0.99 ± 0.19	0.272
Precentral	0.88 ± 0.12	0.91 ± 0.18	0.211
Precuneus	0.96 ± 0.24	1.03 ± 0.34	0.203
Rostral anterior cingulate	0.93 ± 0.22	0.98 ± 0.26	0.261
Rostral middle frontal	0.94 ± 0.27	1.04 ± 0.39	0.110
Superior parietal	1.01 ± 0.19	1.07 ± 0.28	0.166
Superior temporal	1.01 ± 0.14	1.04 ± 0.19	0.382
Supramarginal	1.05 ± 0.20	1.10 ± 0.31	0.259
Inferior temporal	1.08 ± 0.16	1.11 ± 0.24	0.435

References

1. Tustison NJ, Cook PA, Klein A, et al: Large-scale evaluation of ANTs and FreeSurfer cortical thickness measurements. Neuroimage 2014; 99:166-179
2. Avants BB, Epstein CL, Grossman M, et al: Symmetric diffeomorphic image registration with cross-correlation: evaluating automated labeling of elderly and neurodegenerative brain. Med Image Anal 2008; 12:26-41
3. Andersson JLR, Sotiropoulos SN: An integrated approach to correction for off-resonance effects and subject movement in diffusion MR imaging. Neuroimage 2016; 125:1063-1078
4. Garyfallidis E, Brett M, Amirbekian B, et al: Dipy, a library for the analysis of diffusion MRI data. Front Neuroinform 2014; 8:8
5. Muller-Gartner HW, Links JM, Prince JL, et al: Measurement of radiotracer concentration in brain gray matter using positron emission tomography: MRI-based correction for partial volume effects. J Cereb Blood Flow Metab 1992; 12:571-583
6. Erlandsson K, Buvat I, Pretorius PH, et al: A review of partial volume correction techniques for emission tomography and their applications in neurology, cardiology and oncology. Phys Med Biol 2012; 57:R119-159
7. Desikan RS, Segonne F, Fischl B, et al: An automated labeling system for subdividing the human cerebral cortex on MRI scans into gyral based regions of interest. Neuroimage 2006; 31:968-980
8. Mattsson N, Palmqvist S, Stomrud E, et al: Staging beta-amyloid pathology with amyloid positron emission tomography. JAMA Neurol 2019; 76:1319-1329
9. Grothe MJ, Barthel H, Sepulcre J, et al: In vivo staging of regional amyloid deposition. Neurology 2017; 89:2031-2038
10. Levin F, Jelistratova I, Betthauser TJ, et al: In vivo staging of regional amyloid

progression in healthy middle-aged to older people at risk of Alzheimer's disease. Alzheimers Res Ther 2021; 13:178

11. Ota M, Sato N, Nakaya M, et al: Relationships between the deposition of amyloid-beta and tau protein and glymphatic system activity in Alzheimer's disease: diffusion tensor image study. J Alzheimers Dis 2022; 90:295-303
12. Teipel SJ, Kurth J, Krause B, et al: The relative importance of imaging markers for the prediction of Alzheimer's disease dementia in mild cognitive impairment - beyond classical regression. Neuroimage Clin 2015; 8:583-593
13. Battle MR, Pillay LC, Lowe VJ, et al: Centiloid scaling for quantification of brain amyloid with [^{18}F]flutemetamol using multiple processing methods. EJNMMI Res 2018; 8:107
14. Klunk WE, Koeppe RA, Price JC, et al: The Centiloid Project: standardizing quantitative amyloid plaque estimation by PET. Alzheimers Dement 2015; 11:1-15.e1-4
